# Early Dry Eye Disease Onset in a NOD.H-2^h4^ Mouse Model of Sjögren's Syndrome

**DOI:** 10.1167/iovs.63.6.18

**Published:** 2022-06-21

**Authors:** Lili Li, Kimberly J. Jasmer, Jean M. Camden, Lucas T. Woods, Adam L. Martin, Yong Yang, Maria Layton, Michael J. Petris, Olga J. Baker, Gary A. Weisman, Carisa K. Petris

**Affiliations:** 1 Division of Biochemistry, University of Missouri, Columbia, Missouri, United States; 2 Division of Biological Sciences, University of Missouri, Columbia, Missouri, United States; 3 Christopher S. Bond Life Sciences Center, University of Missouri, Columbia, Missouri, United States; 4 Department of Ophthalmology, University of Missouri, Columbia, Missouri, United States; 5 Department of Otolaryngology-Head and Neck Surgery, University of Missouri, Columbia, Missouri, United States; 6 Mason Eye Institute, University of Missouri, Columbia, Missouri, United States; 7 Visual Science and Optometry Center, The People's Hospital of Guangxi Zhuang Autonomous Region, Nanning, Guangxi, China; 8 Department of Ophthalmology, The First Affiliated Hospital of Guangxi Medical University, Nanning, Guangxi, China

**Keywords:** Sjögren's syndrome, dacryoadenitis, sex differences, dry eye, NOD mouse model

## Abstract

**Purpose:**

To develop a mouse model of human dry eye disease (DED) for investigation of sex differences in autoimmune-associated dry eye pathology.

**Methods:**

Ocular surface disease was assessed by quantifying corneal epithelial damage with lissamine green stain in the NOD.H-2^h4^,IFNγ^−^^/^^−^,CD28^−^^/^^−^ (NOD.H-2^h4^ DKO) mouse model of Sjögren's syndrome (SS). Lacrimal gland function was assessed by tear volume quantification with phenol red thread and lacrimal gland inflammation (i.e., dacryoadenitis) was assessed by quantification of immune cell foci, flow cytometric analysis of immune cell composition, and expression of proinflammatory markers.

**Results:**

The NOD.H-2^h4^ DKO mouse model of SS exhibits greater age-dependent increases in corneal damage than in NOD.H-2^h4^ parental mice and demonstrates an earlier disease onset in females compared to males. The severity of ocular surface disease correlates with loss of goblet cell density, increased conjunctivitis, and dacryoadenitis that is more pronounced in NOD.H-2^h4^ DKO than NOD.H-2^h4^ mice. B cells dominate lacrimal infiltrates in 16-week-old NOD.H-2^h4^ and NOD.H-2^h4^ DKO mice, but T helper cells and macrophages are also present. Lacrimal gland expression of proinflammatory genes, including the P2X7 and P2Y_2_ purinergic receptors, is greater in NOD.H-2^h4^ DKO than NOD.H-2^h4^ mice and correlates with dacryoadenitis.

**Conclusions:**

Our results demonstrate for the first time that autoimmune dry eye disease occurs in both sexes of NOD.H-2^h4^ DKO and NOD.H-2^h4^ mice, with earlier onset in female NOD.H-2^h4^ DKO mice when compared to males of the same strain. This study demonstrates that both NOD.H-2^h4^ and NOD.H-2^h4^ DKO mice are novel models that closely resemble SS-related and sex-dependent DED.

Dry eye disease (DED) affects 5% to 50% of the worldwide population,^1^ with an estimated annual cost of $3.84 billion in the United States.^2^ DED is a multifactorial disease resulting in tear film dysfunction and ocular inflammation attributable to insufficient lacrimal gland secretion, meibomian gland dysfunction, and disruption of mucin production by goblet cells.^3^^–^^7^ Additionally, risk factors such as age, sex, medication, and environment may contribute to DED pathogenesis.^7^ Symptoms of DED include discomfort and visual disturbances that can be severe and disabling, resulting in lost work productivity and reduced quality of life.

A common form of DED is attributed to Sjögren's syndrome (SS), a systemic inflammatory autoimmune disease characterized by immune-related dysfunction of lacrimal and salivary glands leading to chronic dry eye and dry mouth, respectively.^8^^–^^12^ SS is typically diagnosed at around 50 years of age and affects ∼4 million individuals in the United States, of whom 90% are females.^8^^–^^10^ Along with dry eye and dry mouth, other hallmarks of SS include the presence of lymphocytes within lacrimal (i.e., dacryoadenitis) and salivary (i.e., sialadenitis) glands, as well as the serum autoantibodies, anti-SSA/Ro and anti-SSB/La.^8^^,^^10^^–^^13^ Lymphocytic foci composed of mainly B and T cells are key mediators of dacryoadenitis and sialadenitis in SS that are associated with chronic hypolacrimation and hyposalivation.^14^ Additionally, DED-associated ocular surface damage in SS is associated with loss of mucin-producing goblet cells, increased conjunctival epithelial inflammation, and meibomian gland dysfunction.^15^^–^^17^ The current study was undertaken to identify a mouse model of SS that exhibits a similar phenotype to early-onset DED in human SS.

The initiating factors in SS are poorly understood. Several mouse models have been generated that exhibit ocular and oral damage resembling human SS^18^^–^^23^; however, these models do not reflect the sexual dimorphism observed in human SS for both ocular and oral damage in a single mouse strain. Among these SS models, the nonobese diabetic (NOD) mouse spontaneously develops sex- and age-dependent lacrimal and salivary gland dysfunction; however, this model is complicated by the presence of non-SS phenotypes such as type-1 diabetes and autoimmune peripheral polyneuropathy.^24^^–^^26^ A nondiabetic variant dubbed NOD.H-2^h4^ was shown to exhibit spontaneous autoimmune thyroiditis^27^^–^^32^ and SS-related disease, including autoantibodies and sialadenitis.^28^^,^^33^^–^^35^ Female NOD.H-2^h4^ mice develop more severe salivary gland disease earlier than males with apparent sialadenitis at ∼12 to 20 weeks of age and salivary gland dysfunction within ∼40 weeks.^28^^,^^33^^–^^35^ Recently, researchers in our group determined that deletion of IFNγ and CD28 in NOD.H-2^h4^ mice (NOD.H-2^h4^ DKO) leads to much earlier onset of autoimmune thyroiditis^36^ and SS-like salivary gland disease,^28^^,^^36^^–^^39^ with severe phenotypes of these pathologies apparent by four months of age. However, it is currently unknown whether either NOD.H-2^h4^ or NOD.H-2^h4^ DKO mice exhibit SS-like ocular pathology similar to human SS and represent good models for early-onset DED in humans. In this study, we show for the first time that DED and dacryoadenitis are present in both male and female NOD.H-2^h4^ mice, a phenotype that has an earlier onset in female NOD.H-2^h4^ DKO mice. The similarity between SS-related ocular disease pathology in NOD.H-2^h4^ and NOD.H-2^h4^ DKO mice and humans suggests that these are ideal mouse models to investigate mechanisms underlying sex-related differences seen with DED in human SS. The early DED onset observed in NOD.H-2^h4^ DKO mice provides a novel model for rapid evaluation of therapeutic interventions to address SS-related DED.

## Materials and Methods

### Mice

BALB/c and NOD.H-2^h4^ mice from Jackson Laboratory (Bar Harbor, ME) and NOD.H-2^h4^ DKO mice from the Mutant Mouse Regional Resource Center at the University of Missouri were housed in a pathogen-free environment for a 12-hour light/dark cycle with free access to standard laboratory chow and water. Development of SS-like DED in both sexes of NOD.H-2^h4^ and NOD.H-2^h4^ DKO mice was assessed at six, 10, 16, and 24 weeks of age. Results showing DED-like features from 16- and 24-week-old mice were equivalent, so only 16-week data are shown. Because there is no isogenic nondiseased control for NOD mice,^26^ age- and sex-matched BALB/c mice were used. Experimental procedures were approved by the University of Missouri Animal Care and Use Committee and conducted in accordance with the Association for Research in Vision and Ophthalmology for the Use of Animals in Ophthalmic and Vision Research and National Institutes of Health guidelines.

### Corneal Staining

Isotonic saline solution (50 µL) was added to a Lissamine Green Ophthalmic Strip (HUB Pharmaceutical, LLC, Plymouth, MI, USA) and 1 µL of the resulting solution was applied to ocular surfaces of anesthetized mice (Avertin 0.75 mg/g mouse weight intraperitoneally) and dispersed by manually blinking the eyelid three times. Corneal epithelial damage/staining was assessed by a blind reviewer using a Stereomaster microscope (ThermoFisher Scientific, Waltham, MA, USA) where a score of 0 is unstained, 1 is <25%, 2 is 25%–50%, 3 is 50%–75%, and 4 is >75% stained.^40^ Cornea photos were obtained using a Leica M205 FA Stereo Microscope (Leica, Wetzlar, Germany).

### Tear Production

Unstimulated tear volume was determined in each eye of anesthetized mice by placing a phenol red thread (ZONE-QUICK, FCI Ophthalmics, Pembroke, MA, USA) in the conjunctival sac at one-third the distance from the lateral canthus of the lower eyelid and gently removing it after 15 seconds. The thread length of the red-stained portion indicates the relative basal tear volume. For stimulated tear volume, 0.25 mg/kg carbachol in saline solution was injected intraperitoneally, and tear production was evaluated three minutes later for 15 seconds.

### Ocular Surface Immunohistochemistry

After cervical dislocation, intact eyes with eyelids were harvested from mice and fixed overnight at 4°C in 4% (v/v) paraformaldehyde in phosphate-buffered saline solution (PBS), washed three times in PBS and placed in 70% (v/v) ethanol for 24 hours. Samples were embedded in paraffin and 5 µm sections were stained with periodic acid-Schiff (PAS) reagent. Goblet cell density continuous with superior and inferior palpebral conjunctiva and conjunctival fornices in two 100 µm PAS-stained sections was determined using a Zeiss Axiovert 200M brightfield microscope with a 10X objective. Sections from 16-week-old mice were stained with anti-CD45 (Biolegend, San Diego, CA) or anti-MMP-9 (Abcam, Cambridge, UK) antibodies. All images were obtained using a Leica DMI6000B brightfield microscope and LASX software.

### Lacrimal Gland Immunohistochemistry

Extraorbital lacrimal glands from six-, 10- and 16-week-old NOD.H-2^h4^ and NOD.H-2^h4^ DKO mice of both sexes were harvested for 5 µm paraffin sectioning and stained with hematoxylin and eosin (H&E). Focus scores were determined from stitched images of lacrimal glands by quantifying lymphocytic foci (>50 mononuclear cells) per 4 mm^2^ tissue. Images were obtained with a Zeiss Axiovert 200M brightfield microscope with a ×10 objective, and the total lacrimal gland area was determined using MetaMorph software. Sections from 16-week-old mice were stained with anti-CD45 or anti-MMP-9 antibodies. Images were obtained using a Leica DMI6000B brightfield microscope and LASX software.

### Flow Cytometry

Lacrimal glands from 16-week-old female and male NOD.H-2^h4^ and NOD.H-2^h4^ DKO mice were minced with scissors in 2 mL of RPMI media plus 2.5 mM CaCl_2_ and 4 mg/mL Collagenase D (Sigma Aldrich, St. Louis, MO, USA) and shaken at 250 rpm at 37°C for one hour. Dispersed cells were passed through a 40 µm nylon filter and rinsed with 8 mL of PBS. Cells were pelleted at 400 *g* for five minutes and re-suspended in 5 mL of red blood cell lysis buffer (Miltenyi Biotec, Auburn, CA, USA) for 15 minutes in the dark. Samples were pelleted (400 *g*; five minutes) and re-suspended in 1 mL of PBS as a single-cell suspension for flow cytometry. Leukocytes were classified using Miltenyi Biotec anti-mouse fluorophore-conjugated antibodies for: CD45 (VioBlue; pan-leukocyte marker), F4/80 (FITC; macrophages), CD4 (PE-Vio615; T helper cells), CD19 (PE-Vio770; B cells), CD8a (APC-Vio770; cytotoxic T cells) and CD3 (PerCP-Cy5.5; pan-T cell marker; BioLegend, San Diego, CA, USA). VioBility 405/520 fixable viability dye (Miltenyi Biotec) identified dead cells. Data were obtained using a BD Fortessa X20 flow cytometer (University of Missouri Cell and Immunobiology Core facility), analyzed with FlowJo software (v10.4.2), and expressed as a percentage of CD45^+^ cells. The gating strategy used is included in the Supplementary Materials, showing representative spleen and lacrimal gland data.

### Real-Time PCR

Lacrimal glands from 16-week-old NOD.H-2^h4^, NOD.H-2^h4^ DKO, and BALB/c mice of both sexes were homogenized in TRIzol reagent (ThermoFisher Scientific), chloroform (0.2 mL/ml H_2_O) was added, mixed vigorously, incubated for five minutes at room temperature, and spun in centrifuge at 12,000 *g* for five minutes at 4°C. Total RNA was extracted from the resulting aqueous phase using the RNeasy Plus Micro kit (Qiagen, Valencia, CA, USA), and cDNA was prepared from the RNA (1 µg) using RNA to cDNA EcoDry Premix (Takara Bio, Mountain View, CA, USA). Quantitative RT-PCR was performed on an Applied Biosystems 7500 real-time PCR machine using specific Taqman primers for mouse IL-1β, IL-2, IL-6, B cell-activating factor (BAFF), TNF-α, ICAM-1, and P2X7 and P2Y_2_ receptors (Applied Biosystems, Foster City, CA); 18S ribosomal RNA was used as an internal control and data were analyzed using Applied Biosystems software.

### Statistical Analyses

Statistical analyses were performed with GraphPad Prism 9.0 software and data represent means ± SEM from at least three independent experiments where *P* values < 0.05 were considered significant. Two- or three-way ANOVA followed by either Tukey's or uncorrected Fisher's LSD multiple comparisons tests, or unpaired Student's *t*-test for two-group comparison of parametric data, were used as indicated in the figure legends. For correlation analyses, Pearson correlations were conducted, and r and *P* values were reported.

## Results

### Both Male and Female NOD.H-2^h4^ and NOD.H-2^h4^ DKO Mice Exhibit Ocular Surface Damage

**Figure 1.**
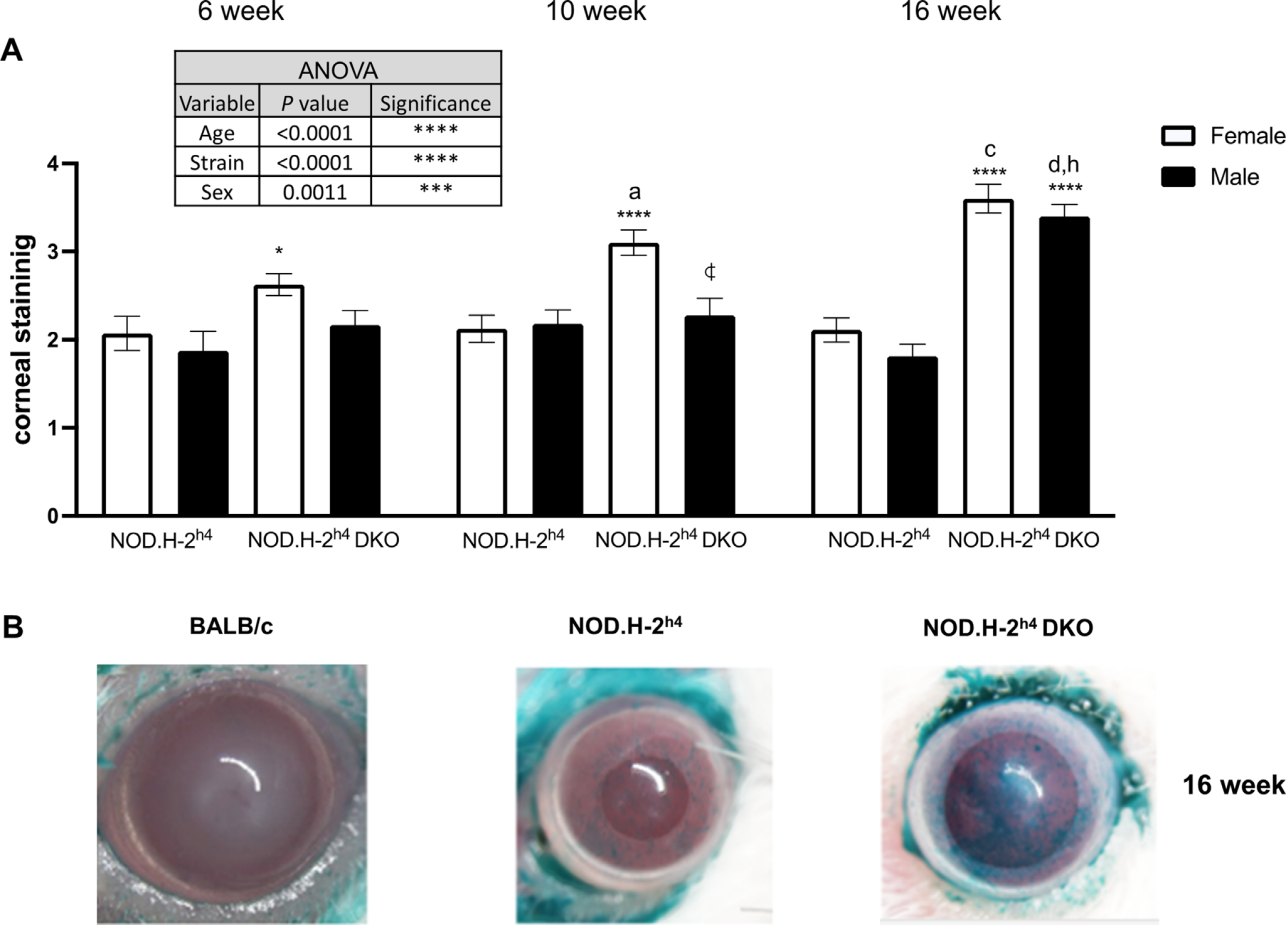
Corneal damage exhibited by NOD.H-2^h4^ and NOD.H-2^h4^ DKO mice demonstrates age, strain, and sex differences. **(A)** Lissamine green corneal staining in anesthetized six-, 10- and 16-week-old female and male NOD.H-2^h4^ and NOD.H-2^h4^ DKO mice. Staining was quantified as described in Materials and Methods. Data represent means ± SEM for 10 to 20 mice. Three-way ANOVA was conducted and statistically significant differences between strains, age, and sex of NOD.H-2^h4^ DKO and NOD.H-2^h4^ mice are shown in the *inset table*. ***P < 0.001; ****P < 0.0001. Post-ANOVA uncorrected Fisher's LSD multiple comparisons test identified statistically significant differences, where 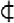 indicates *P <* 0.001 compared to the opposite sex for the same age and strain, *a*, *c*, and *d* indicate *P* < 0.05, *P* < 0.001, and *P* < 0.0001, respectively, compared to six-week-old mice of the same sex and strain, *h* indicates *P* < 0.0001 compared to 10-week-old mice of the same sex and strain, and * and **** indicate *P* < 0.05 and *P* < 0.0001, respectively, for NOD.H-2^h4^ DKO mice compared to NOD.H-2^h4^ mice of the same age and sex. **(B)** Representative corneal staining in 16-week-old female BALB/c, NOD.H-2^h4,^ and NOD.H-2^h4^ DKO mice.

Ocular surface staining is an important clinical parameter used to determine DED in SS patients.^41^^,^^42^ Lissamine green staining highlights damaged corneal epithelium and devitalized cells to which it binds on the ocular surface.^42^ Using this approach, we determined that both sexes of six-, 10-, and 16-week-old NOD.H-2^h4^ and NOD.H-2^h4^ DKO mice exhibit ocular surface damage (Figs. 1A, 1B), whereas no appreciable staining was observed in female (Fig. 1B) or male (not shown) BALB/c mice. Three-way ANOVA revealed age-, sex-, and strain-dependent differences among corneal staining data. No significant differences in corneal staining were observed between male and female NOD.H-2^h4^ mice at any age tested (Fig. 1A); however, staining in 10-week-old female NOD.H-2^h4^ DKO mice was significantly higher than age-matched males (Fig. 1A). Female NOD.H-2^h4^ DKO mice had significantly greater corneal staining than age-matched female NOD.H-2^h4^ mice at all ages tested, whereas this increase was only seen with 16-week-old male NOD.H-2^h4^ DKO mice (Fig. 1A). Although both male and female NOD.H-2^h4^ and NOD.H-2^h4^ DKO mice exhibit corneal damage, both sexes of NOD.H-2^h4^ DKO mice display significantly more corneal damage than sex-matched NOD.H-2^h4^ mice at 16 weeks of age. Neither sex of NOD.H-2^h4^ mice displayed age-dependent increases in corneal damage whereas corneal staining scores increased by 28% in female and 37% in male NOD.H-2^h4^ DKO mice from six to 16 weeks of age.

### Both Male And Female NOD.H-2^h4^ and NOD.H-2^h4^ DKO Mice have Reduced Goblet Cell Densities

**Figure 2.**
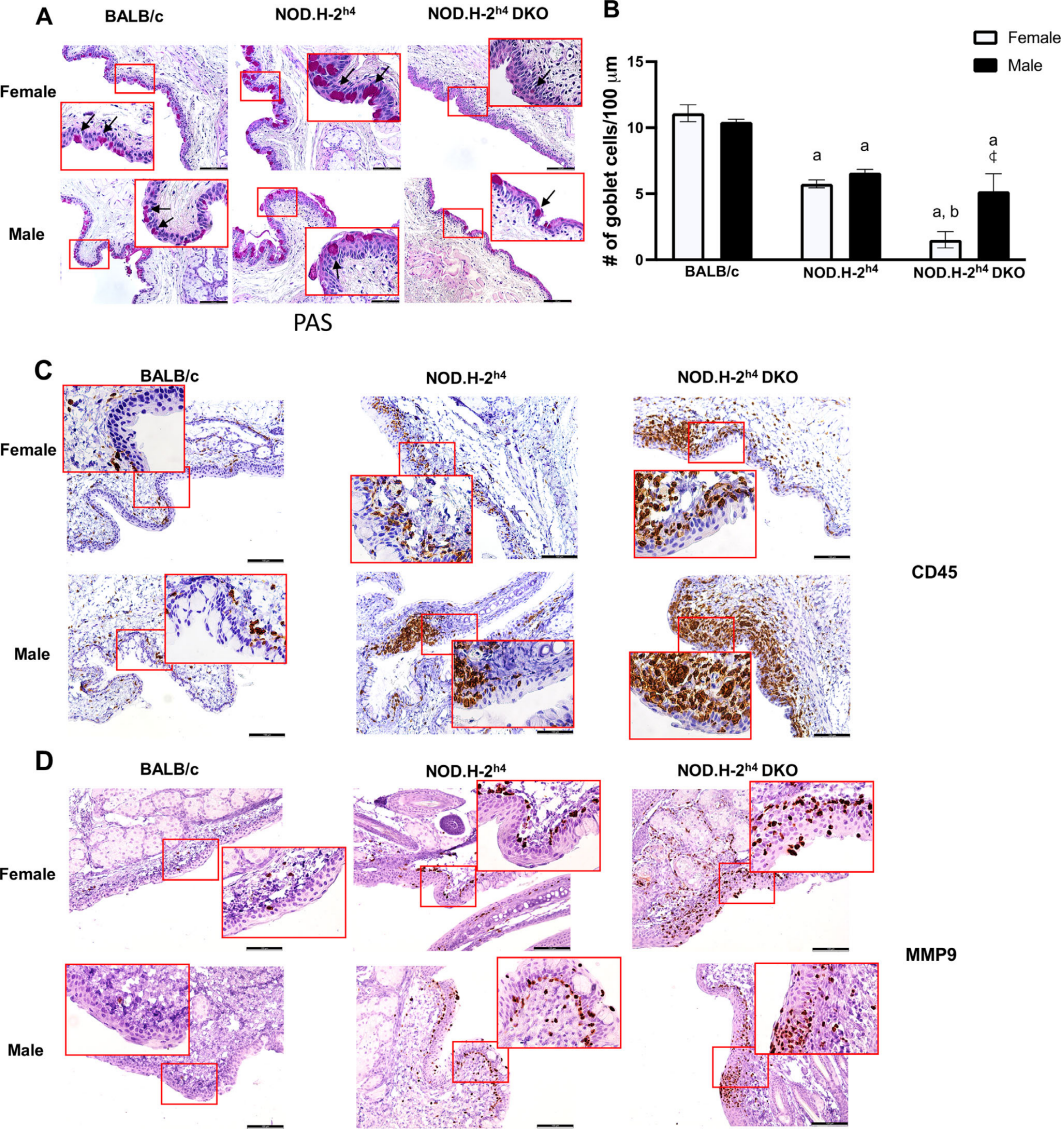
Goblet cell density is significantly reduced to a greater extent in 16-week-old female NOD.H-2^h4^ DKO mice than in age-matched male NOD.H-2^h4^ DKO mice or NOD.H-2^h4^ mice of both sexes, as compared to control BALB/c mice. **(A)** Representative PAS staining of the conjunctiva of 16-week-old female and male NOD.H-2^h4^, NOD.H-2^h4^ DKO, and BALB/c mice (magnification ×200; *scale* *bar:* 100 µm; **inset:** image ×2). Goblet cells are indicated with *black arrows*. **(B)** Goblet cell density (cells per 100 µm). Data represent means ± SEM for five to 10 mice. Two-way ANOVA was conducted followed by Tukey's (strain) or Fisher's LSD (sex) multiple comparison tests, where *a* and *b* indicate *P* < 0.0001 compared to BALB/c and NOD.H-2^h4^ mice, respectively, and 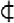 indicates *P* < 0.001 compared to females of the same strain. **(C)** Representative immunohistochemical images of CD45^+^ leukocytic infiltrates in mouse conjunctiva (magnification ×100; *scale* *bar:* 100 µm; **inset:** image ×2). **(D)** Representative immunohistochemical images of MMP-9 staining in mouse conjunctiva (magnification ×100; *scale* *bar:* 100 µm; **inset:** image ×2).

A potential cause of the observed corneal and ocular surface damage is the loss of mucin-producing goblet cells.^15^ We investigated goblet cell abundance in the fornix conjunctiva of NOD.H-2^h4^ and NOD.H-2^h4^ DKO mice at 16 weeks when DED is readily apparent. Results show that goblet cell density is significantly decreased in forniceal conjunctiva of male and female NOD.H-2^h4^ and NOD.H-2^h4^ DKO mice when compared to age- and sex-matched BALB/c controls (Figs. 2A, 2B). No significant differences in goblet cell density scores were observed between male and female NOD.H-2^h4^ mice; however, female NOD.H-2^h4^ DKO mice had significantly lower goblet cell density than either sex of NOD.H-2^h4^ mice and male NOD.H-2^h4^ DKO mice (Figs. 2A, 2B).

Lymphocytic infiltration of the conjunctiva has been reported in both SS patients and murine models of SS.^43^^–^^45^ Compared to BALB/c mice, CD45 staining in both sexes of NOD.H-2^h4^ and NOD.H-2^h4^ DKO mice showed an abundance of immune cells throughout the conjunctiva (Fig. 2C). Matrix metalloprotease-9 (MMP-9) has been used as an objective diagnostic measure of ocular surface inflammation in DED.^46^^,^^47^ Although we found no MMP-9 staining on the corneal surface of any of the mouse strains of both sexes (not shown), we observed MMP-9 staining in the conjunctiva of NOD.H-2^h4^ and NOD.H-2^h4^ DKO but little in BALB/c mice (Fig. 2D). MMP-9 staining was greatest in the conjunctiva of both sexes of NOD.H-2^h4^ DKO mice as compared to NOD.H-2^h4^ mice (Fig. 2D). These data suggest that reduced goblet cell density, the presence of immune cell infiltrates, and enhanced MMP-9 expression in the conjunctiva are markers of ocular damage underlying DED in these mouse models of SS.

### Increased Dacryoadenitis Severity in Male and Female NOD.H-2^h4^ DKO Mice

**Figure 3.**
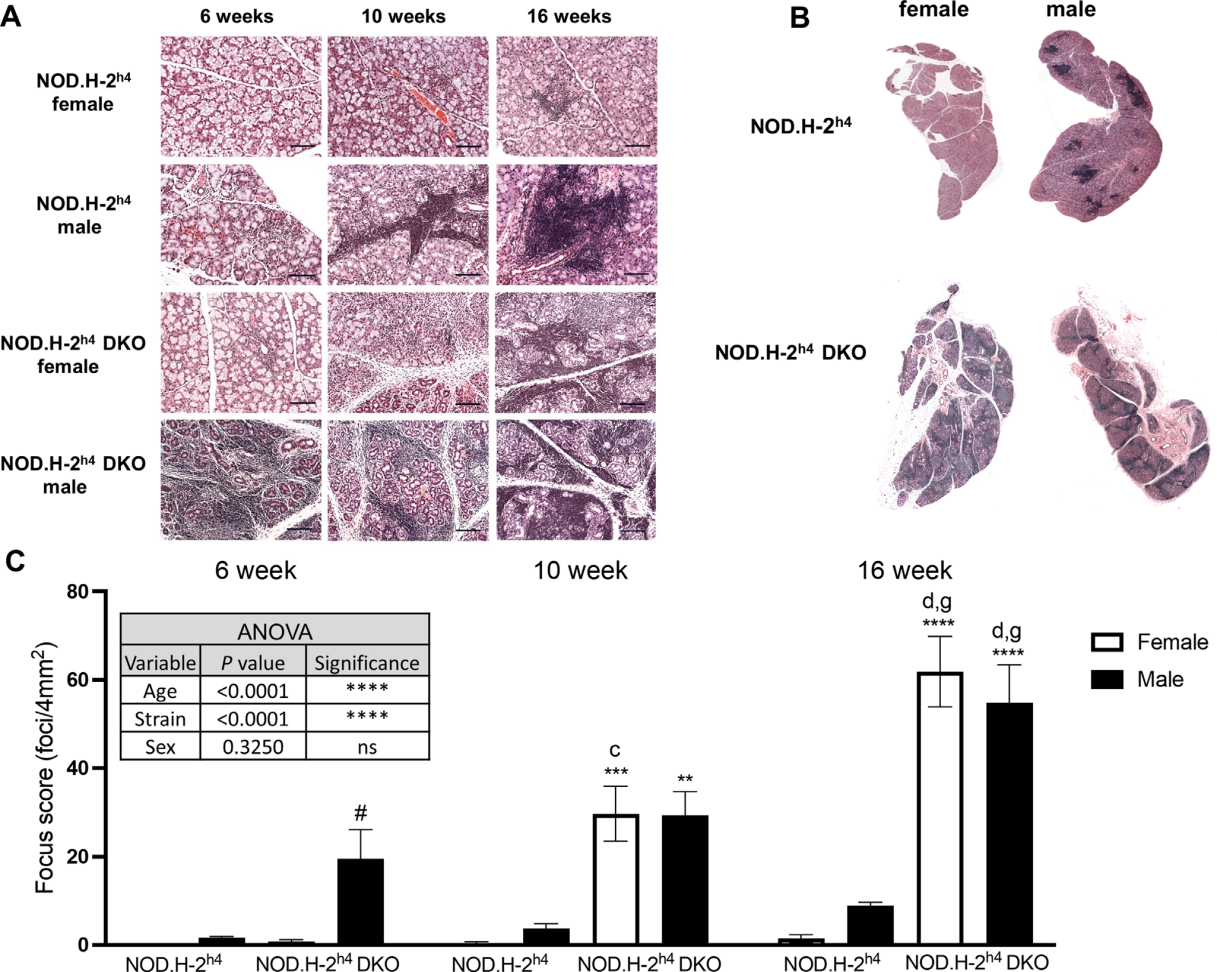
Early-onset dacryoadenitis development in NOD.H-2^h4^ DKO mice. **(A)** Representative H&E staining in extraorbital lacrimal gland sections (magnification ×200; *scale* *bar:* 100 µm) from six-, 10-, and 16-week-old female and male NOD.H-2^h4^ and NOD.H-2^h4^ DKO mice. **(B)** H&E-stained whole gland sections (magnification ×50) from 16-week-old female and male mice. **(C)** Lacrimal gland focus scores (lymphocytic foci/4 mm^2^ gland area). Data represent means ± SEM for six to eight mice. Three-way ANOVA was conducted and statistically significant differences between strains, age, and sex for NOD.H-2^h4^ DKO and NOD.H-2^h4^ mice are shown in the **inset table**. ****P < 0.0001; ns, no significance. Post-ANOVA uncorrected Fisher's LSD multiple comparisons test identified statistically significant differences, where # indicates *P <* 0.05 compared to the opposite sex of the same age and strain, *c* and *d* indicate *P* < 0.001 and *P* < 0.0001, respectively, compared to six-week-old mice of the same sex and strain, *g* indicates *P* < 0.001 compared to 10-week-old mice of the same sex and strain, and **, ***, and **** indicate *P* < 0.01, *P* < 0.001 and *P* < 0.0001, respectively, for NOD.H-2^h4^ DKO mice compared to NOD.H-2^h4^ mice of the same age and sex.

Studies report that male NOD-derived mice acquire more severe dacryoadenitis than age-matched females.^48^^,^^49^ Histological examinations show significant age-dependent increases in immune cell infiltration of H&E-stained lacrimal gland sections in both sexes of NOD.H-2^h4^ DKO mice compared to NOD.H-2^h4^ mice, and male NOD.H-2^h4^ DKO mice at six weeks of age show greater dacryoadenitis than females (Figs. 3A–C). These data indicate that male NOD.H-2^h4^ DKO mice exhibit earlier-onset autoimmune dacryoadenitis than females, whereas both sexes show significantly more rapid progression of severe dacryoadenitis over 16 weeks as compared to NOD.H-2^h4^ mice.

### Reduced Tear Production in Male and Female NOD.H-2^h4^ DKO Mice

**Figure 4.**
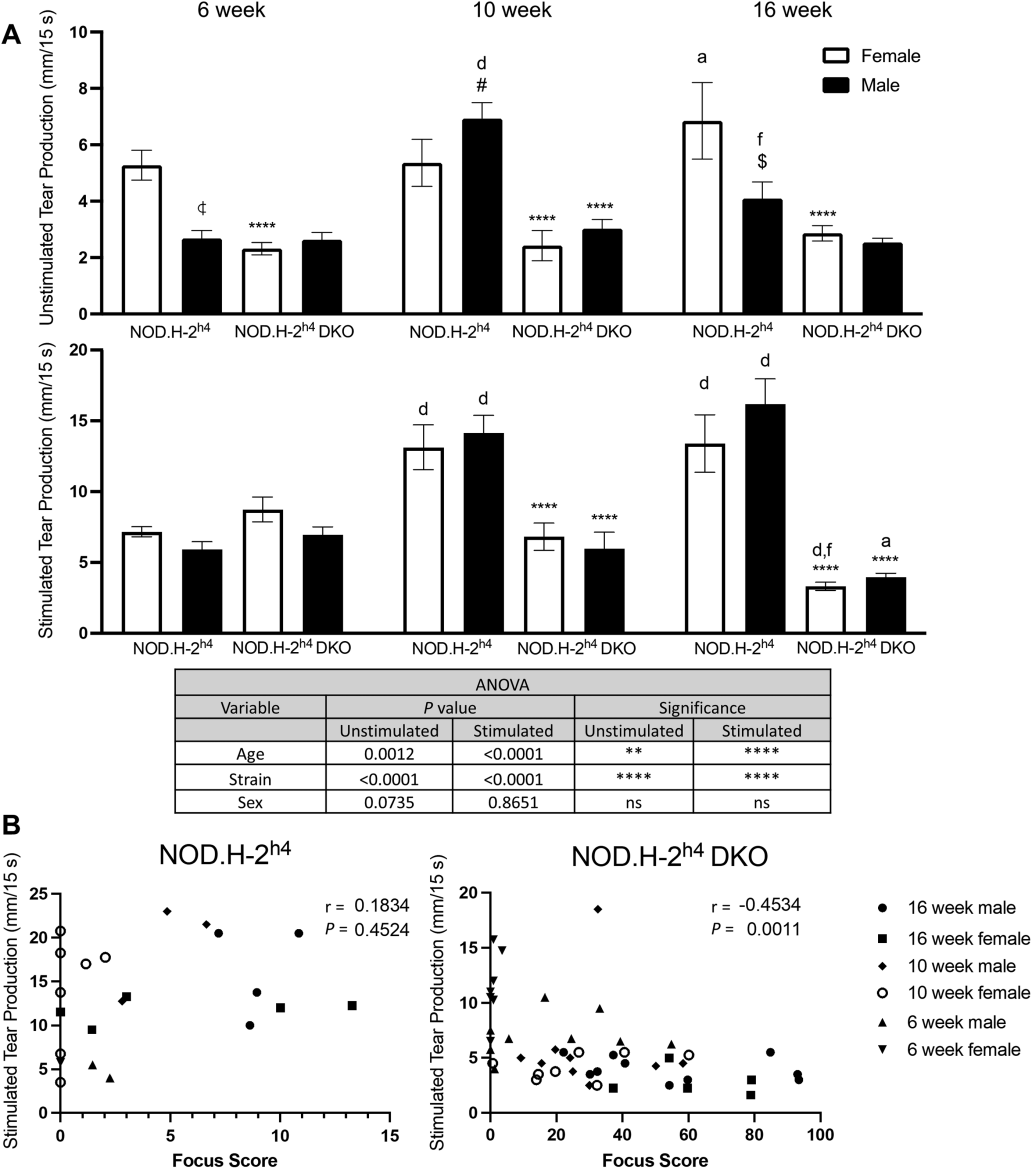
Reduced tear production in NOD.H-2^h4^ DKO mice. **(A)** Quantitative assessment of unstimulated (top) and 0.25 mg/kg carbachol-stimulated (bottom) tear production (mm/15 s) was performed using phenol red cotton thread, as described in *Materials and Methods*, in six-, 10- and 16-week-old female and male NOD.H-2^h4^ and NOD.H-2^h4^ DKO mice. Data represent means ± SEM for eight to 20 mice. Three-way ANOVA was conducted, and significant differences between age, strain, and sex are shown in the **inset table**. **P < 0.01; ****P < 0.0001; ns, no significance. Post-ANOVA uncorrected Fisher's LSD multiple comparisons test identified statistically significant differences, where #, $, and 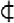 indicate *P <* 0.05, *P <* 0.01, and *P <* 0.001, respectively, compared to the opposite sex of the same age and strain, *a* and *d* indicate *P* < 0.05 and *P* < 0.0001, respectively, compared to six-week-old mice of the same sex and strain, *f* indicates *P* < 0.01 compared to 10-week-old mice of the same sex and strain, and **** indicates *P* < 0.0001 for NOD.H-2^h4^ DKO mice compared to NOD.H-2^h4^ DKO mice of the same age and sex. **(B)** Pearson correlation reveals an inverse relationship between the volume of stimulated tear production and lymphocytic focus score in NOD.H-2^h4^ DKO mice (*right panel*) but not in NOD.H-2^h4^ mice (*left panel*). The r and *P* values for each correlation analysis are displayed on the graphs.

Loss of lacrimal gland function occurs in SS mouse models, but the loss of tear production does not correlate directly with glandular inflammation in all models.^49^^,^^50^ Therefore we assessed lacrimal gland tear secretion by measuring unstimulated and carbachol-stimulated tear production (Fig. 4A). Unstimulated and stimulated tear production was significantly reduced to a similar extent in both sexes of 10- and 16-week-old NOD.H-2^h4^ DKO mice as compared to NOD.H-2^h4^ mice of the same age and sex (Fig. 4A), which correlates with the times when significant increases in lymphocytic foci are detected in NOD.H-2^h4^ DKO mice (Fig. 3). Unstimulated tear production in six- and 16-week-old male NOD.H-2^h4^ mice was significantly lower than in age-matched females (Fig. 4), whereas lymphocytic foci are not present in these males until 10 weeks. Compared to six weeks of age, stimulated tear production at 10 and 16 weeks in both sexes of NOD.H-2^h4^ mice was increased (Fig. 4A). In contrast, stimulated tear production in both male and female NOD.H-2^h4^ DKO mice was significantly reduced at 16 compared to six weeks of age (Fig. 4A). Significant age- and strain-dependent differences in unstimulated and stimulated tear production occur, but sex-related differences were insignificant (Fig. 4A, inset). Pearson correlation analysis revealed a significant inverse relationship between tear production and lymphocytic foci in the NOD.H-2^h4^ DKO mice of both sexes but not in NOD.H-2^h4^ mice (Fig. 4B). Thus loss of tear production correlates with early dacryoadenitis in NOD.H-2^h4^ DKO mice of both sexes, suggesting that a potential link between lacrimal gland inflammation and loss of tear secretion requires further investigation in this SS mouse model.

It has previously been reported that NOD.H-2^h4^ mice produce detectable anti-SSA/Ro and anti-SSB/La autoantibodies, although their levels at ≤16 weeks, as used in this study, were very low.^34^ We were unable to detect anti-SSA/Ro and anti-SSB/La autoantibodies in six- to 16-week-old NOD.H-2^h4^ DKO mouse serum by commercial ELISA (not shown).

### Immune Cell Populations in Lacrimal Glands of NOD.H-2^h4^ and NOD.H-2^h4^ DKO Mice

**Figure 5.**
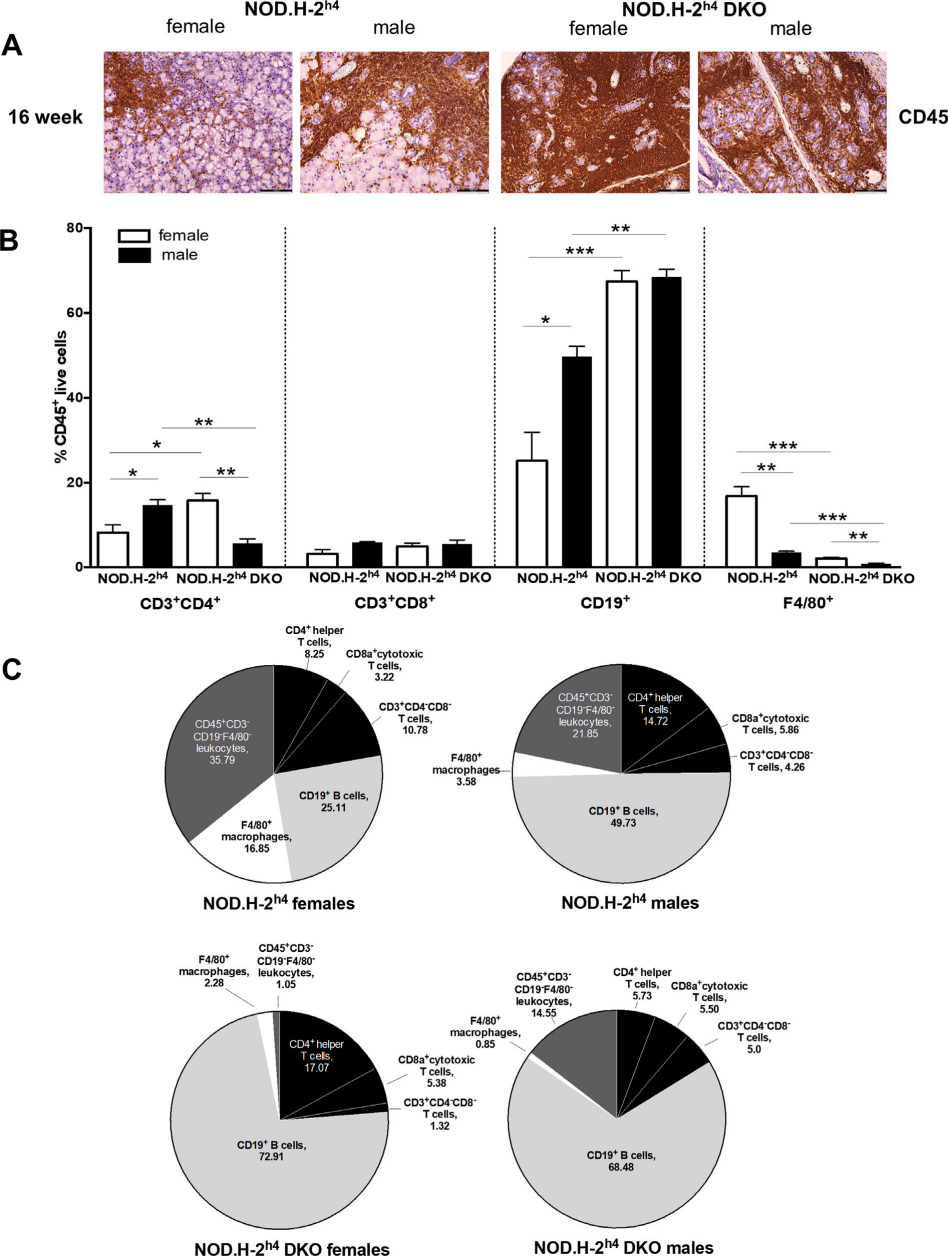
Analysis of immune cell populations in lacrimal glands of NOD.H-2^h4^ and NOD.H-2^h4^ DKO mice. **(A)** Representative paraffin-embedded lacrimal gland sections from 16-week-old female and male NOD.H-2^h4^ and NOD.H-2^h4^ DKO mice were subjected to immunohistochemical analysis for pan-leukocyte antigen CD45^+^. **(B)** Quantitative flow cytometry of immune cells from lacrimal glands of 16-week-old female and male NOD.H-2^h4^ and NOD.H-2^h4^ DKO mice stained with fluorophore-conjugated antibodies to CD45, CD3 (general T cell marker), CD4 (T helper cell marker), CD8 (cytotoxic T cell marker), CD19 (B cell marker) and F4/80 (macrophage marker). Data are shown as a percentage of CD45^+^ live cells. Data represent means ± SEM for six to 10 mice. Statistical significance was determined by standard two-tailed Student's *t*-test: *P < 0.05, **P < 0.01, and ***P < 0.001. **(C)** Pie charts of percentages of immune cell populations in lacrimal glands of 16-week-old mice.

Salivary and lacrimal gland infiltrates of SS patients^51^^,^^52^ and NOD mice^53^^,^^54^ include CD4^+^ T helper cells, B cells, macrophages, dendritic cells, and other immune cells. Flow cytometric analysis of CD45^+^ immune cells isolated from the lacrimal glands of 16-week-old female and male NOD.H-2^h4^ and NOD.H-2^h4^ DKO mice (Fig. 5A) indicates that CD19^+^ B cells are the predominant immune cell infiltrate in lacrimal glands of both strains of mice (Figs. 5B, 5C). A significantly higher percentage of CD19^+^ B cells and CD3^+^CD4^+^ T helper cells and a lower percentage of F4/80^+^ macrophages are present in the lacrimal glands of male compared to female NOD.H-2^h4^ mice (Figs. 5B, 5C). Female NOD.H-2^h4^ DKO mouse lacrimal glands show higher levels of CD3^+^CD4^+^ and F4/80^+^ cells than males, whereas CD19^+^ B cell levels were similar for both sexes (Figs. 5B and 5C). Compared to age- and sex-matched NOD.H-2^h4^ mice, CD19^+^ B cells in both male and female NOD.H-2^h4^ DKO mice were more abundant, whereas CD3^+^CD4^+^ cells were higher in females and lower in males (Figs. 5B and 5C).

### Increased Expression of Proinflammatory Markers in Lacrimal Glands of Male and Female NOD.H-2^h4^ DKO Mice

**Figure 6.**
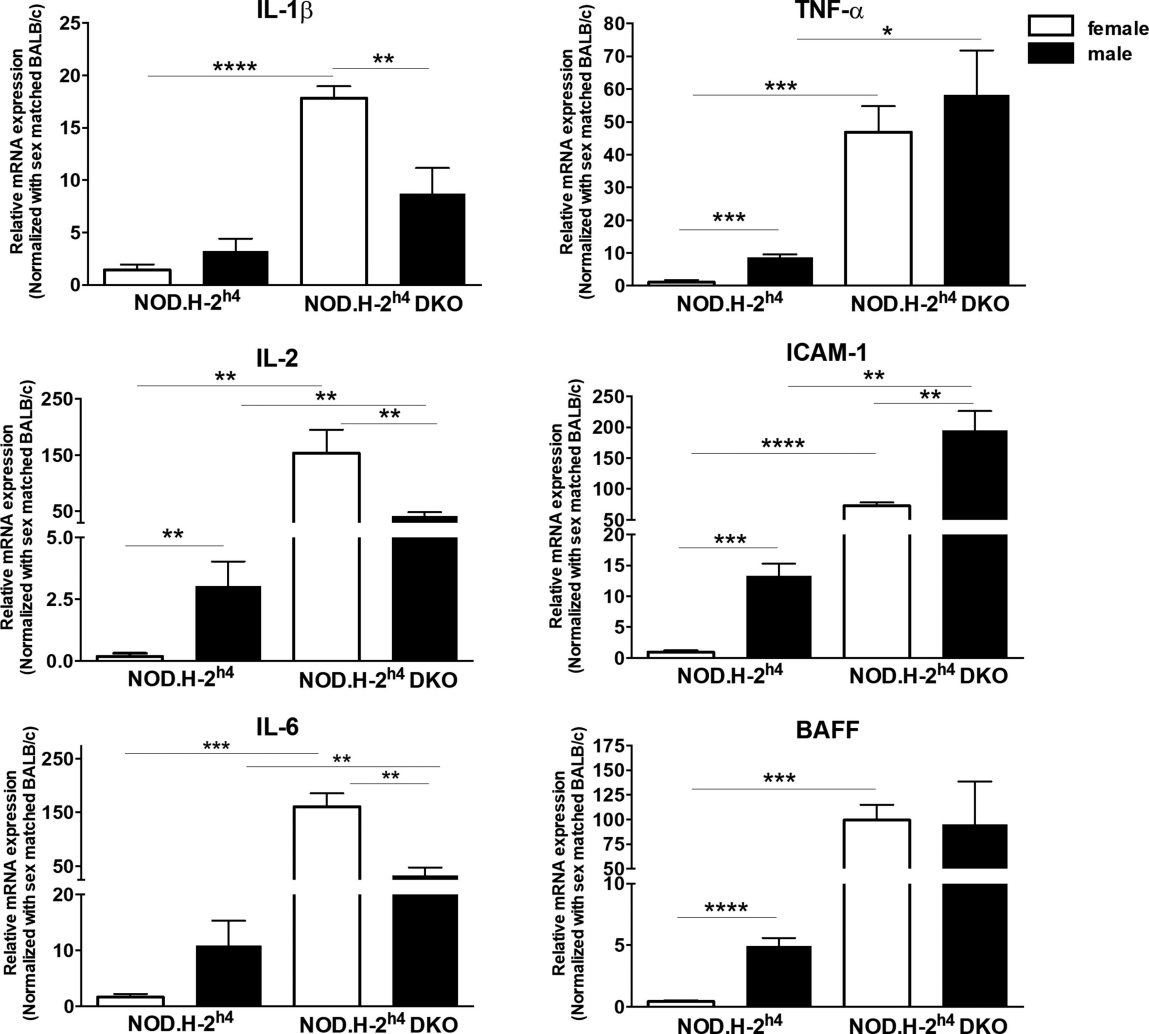
Expression of proinflammatory markers in lacrimal glands of male and female NOD.H-2^h4^ and NOD.H-2^h4^ DKO mice. Quantitative RT-PCR of cDNA from mRNA isolated from the lacrimal glands of 16-week-old female and male NOD.H-2^h4^ and NOD.H-2^h4^ DKO mice was used to measure expression levels of the proinflammatory genes *IL-1β*, *IL-2*, *IL-6*, *TNF-α*, *ICAM-1* and *BAFF*, normalized to sex- and age-matched BALB/c mouse cDNA. Data represent means ± SEM for three to six mice. *P < 0.05, **P < 0.01, ***P < 0.001, ****P < 0.0001.

Compared to age- and sex-matched NOD.H-2^h4^ mice, the lacrimal glands of both 16-week-old female and male NOD.H-2^h4^ DKO mice exhibit significantly higher levels of mRNAs corresponding to the proinflammatory genes IL-2, IL-6, TNF-α, and ICAM-1, whereas NOD.H-2^h4^ DKO females also showed elevated levels of proinflammatory IL-1β and BAFF (Fig. 6). However, a comparison of sex differences within NOD.H-2^h4^ mice revealed that the expression of IL-2, TNF-α, BAFF, and ICAM-1 were significantly higher in the lacrimal glands of males compared to females (Fig. 6). In NOD.H-2^h4^ DKO mice, the lacrimal glands of females exhibited elevated IL-1β, IL-2, and IL-6 and decreased ICAM-1 expression relative to males (Fig. 6). These data correlate with the magnitude of dacryoadenitis in the lacrimal glands of these mice (Fig. 3). BALB/c mouse lacrimal glands showed low expression levels of these proinflammatory genes (not shown).

**Figure 7.**
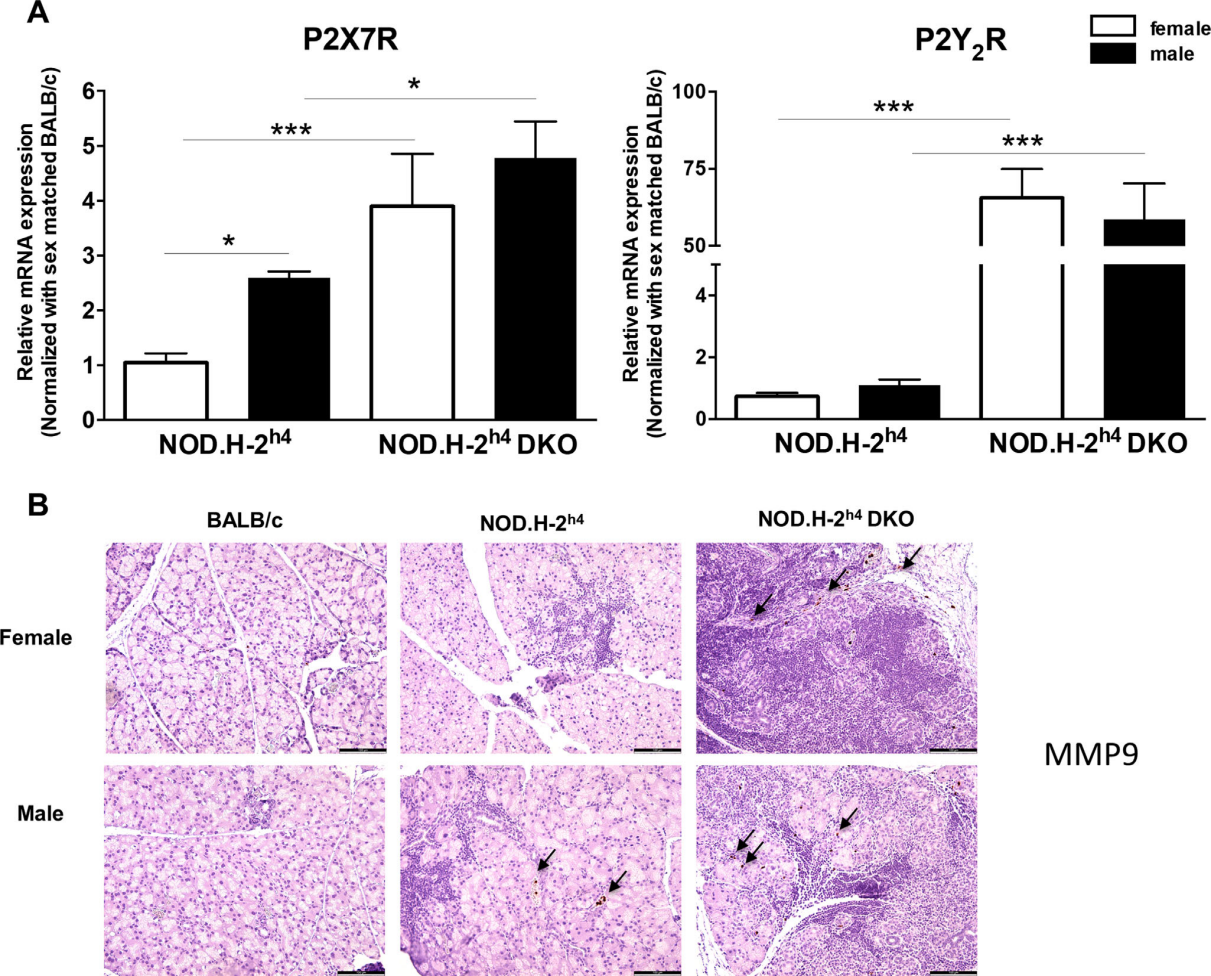
Expression of P2X7 and P2Y_2_ receptor mRNA in lacrimal glands of NOD.H-2^h4^ and NOD.H-2^h4^ DKO mice. **(A)** Quantitative RT-PCR of cDNA from lacrimal gland mRNA of 16-week-old female and male NOD.H-2^h4^ and NOD.H-2^h4^ DKO mice was used to measure *P2X7R* and *P2Y_2_R* expression, normalized to sex-matched BALB/c mouse cDNA. Data represent means ± S.E.M. for 3-6 mice, where * and *** indicate *P* < 0.05 and *P* < 0.001, respectively. **(B)** Representative immunohistochemical images of MMP-9 staining in lacrimal glands from 16-week-old mice (magnification ×100; *scale* *bar:* 100 µm).

Expression of the P2X7 receptor (P2X7R) and the P2Y_2_ receptor (P2Y_2_R) for extracellular adenosine 5ʹ-triphosphate are upregulated under proinflammatory conditions,^55^^–^^60^ including in salivary glands of SS patients,^61^ mouse models of SS,^62^^,^^63^ and activated immune cells.^64^^–^^67^ At 16 weeks, the mRNA levels of both P2X7Rs and P2Y_2_Rs are upregulated in lacrimal glands of both sexes of NOD.H-2^h4^ DKO mice compared to sex-matched NOD.H-2^h4^ mice (Fig. 7A). P2X7R expression in the lacrimal glands of male NOD.H-2^h4^ mice was elevated relative to females (Fig. 7A). P2X7R and P2Y_2_R upregulation in the lacrimal glands of NOD.H-2^h4^ DKO compared to NOD.H-2^h4^ mice correlates with increased levels of lymphocytic foci in the lacrimal glands of NOD.H-2^h4^ DKO mice relative to age- and sex-matched NOD.H-2^h4^ mice (Fig. 3). We also observed MMP-9 staining in the lacrimal glands of NOD.H-2^h4^ DKO mice of both sexes (Fig. 7B). Because P2X7R-mediated IL-1β release upregulates P2Y_2_R^57^ and MMP-9,^68^^,^^69^ the demonstrated increase in IL-1β levels in NOD.H-2^h4^ DKO mouse lacrimal glands (Fig. 6) likely regulates increased P2Y_2_R and MMP-9 expression (Fig. 7). In this way, P2X7R expression and activation may promote DED through multiple mechanisms in NOD.H-2^h4^ DKO mice.

## Discussion

Previous studies show that multiple factors contribute to DED in SS including tear film instability, decreased goblet cell density, and conjunctival inflammation.^9^^,^^13^^,^^16^^,^^70^ An optimal mouse model of SS-like DED that acquires ocular manifestations mimicking the pathogenesis of human DED within a time frame that faithfully recapitulates clinical manifestations in humans both with regard to female-dominance and simultaneous ocular and oral symptom severity would have significant relevance to the use of mice for investigating underlying cellular mechanisms in human SS pathogenesis that will promote novel therapeutic interventions currently lacking. NOD-derived mouse strains have a decreased density of conjunctival goblet cells that secrete mucins to maintain ocular surface homeostasis,^71^ correlating with ocular surface damage and conjunctival inflammation.^72^ Although nonobese diabetes-resistant (NOR) mice are also a diabetes-resistant NOD strain, similar to NOD.H-2^h4^ mice, nonobese diabetes-resistant mice are considered to be a poor model for autoimmune dacryoadenitis.^73^ The current study indicates that male NOD.H-2^h4^ mice exhibit SS-like autoimmune dacryoadenitis (Fig. 3), and demonstrates that male and female NOD.H-2^h4^ DKO mice exhibit early dacryoadenitis (Fig. 3) and reduced tear secretion (Fig. 4) to a greater extent than sex- and age-matched NOD.H-2^h4^ mice. Corneal damage increases with age in NOD.H-2^h4^ DKO mice, and importantly, is more pronounced in females than males (Fig. 1A), which correlates with increased conjunctival inflammation (Fig. 3) and loss of goblet cell density (Figs. 2A, 2B). Because suppression of IFN-γ alone preserves goblet cells,^71^ the significant loss of goblet cell density in the NOD.H-2^h4^ DKO mice with IFN-γ knockout was unexpected. However, deletion of both IFN-γ and CD28 in T cells of NOD.H-2^h4^ DKO mice may interfere with cell survival signals, leading to earlier onset ocular disease, similar to thyroiditis^28^^,^^34^ and sialadenitis^28^^,^^37^ in this mutant strain. There is also another possibility that might explain this phenomenon. Using the RXRα Pinkie mouse model, which develops many characteristics of DED including goblet cell loss, it was recently reported that IL-17+ γδ T cells found in the conjunctiva contribute to MMP-9 production and goblet cell loss, both of which could be blocked with an anti-IL-17 monoclonal antibody.^74^ IL-17 is an important contributor to SS pathogenesis.^75^^–^^77^ Additionally, activation of P2X7R, which is elevated in the lacrimal glands of NOD.H-2^h4^ DKO mice (Fig. 7) and contributes to goblet cell function,^78^ has been shown to increase levels of IL-17A production and secretion.^79^^–^^81^ Future studies will investigate mechanisms by which the deletion of IFN-γ and CD28 results in goblet cell loss and exacerbated DED in the NOD.H-2^h4^ DKO mouse model of SS.

Lacrimal gland dysfunction is known to contribute to loss of tear production.^7^^,^^9^^–^^11^ We found that both unstimulated and carbachol-stimulated tear secretion were significantly reduced in NOD.H-2^h4^ DKO mice compared to sex- and age-matched NOD.H-2^h4^ mice (Fig. 4). Although NOD.H-2^h4^ mice show insignificant increases in dacryoadenitis with age (Fig. 3) with a concomitant increase in stimulated tear secretion (Fig. 4A), NOD.H-2^h4^ DKO mice demonstrate robust increases in dacryoadenitis with age (Fig. 3) and corresponding decreases in stimulated tear production (Fig. 4). Increased carbachol-stimulated tear production with age in NOD.H-2^h4^ mice may be due to ocular irritation,^82^ as seen in Figures 1 and 2, or alterations in tear composition.^49^ Androgens also have been implicated in lacrimal gland autoimmunity,^83^^,^^84^ although both sexes of NOD.H-2^h4^ DKO mice exhibit early-onset dacryoadenitis (Fig. 3) and sialadenitis^36^ that is relatively unique for a mouse model of DED. To our knowledge, this is the first NOD-derived mouse where females exhibit the same extent of dacryoadenitis as males, thus more effectively reproducing the proper sex distribution of human SS, making the NOD.H-2^h4^ DKO mouse model ideal for investigating both salivary and lacrimal gland dysfunction in a single strain.

Lymphocytic infiltration of lacrimal and salivary glands is a hallmark of SS, where B and T cells, macrophages, and dendritic cells contribute to glandular inflammation.^12^^,^^14^ Initially, CD4^+^ T helper cell infiltration precedes the accumulation of B cells,^53^^,^^54^ eventually forming B cell-centered foci surrounded by T cells.^49^ Lacrimal glands isolated from male and female NOD.H-2^h4^ DKO mice had higher CD19^+^ B cell percentages than those collected from NOD.H-2^h4^ mice, whereas CD3^+^CD4^+^ T helper cells were higher in female than male NOD.H-2^h4^ DKO mice (Figs. 5B, 5C). Although CD8^+^ cytotoxic T cells contribute to lacrimal gland pathology,^85^ their numbers do not vary with disease progression in SS mouse models^85^^,^^86^ nor do they vary between lacrimal glands of NOD.H-2^h4^ and NOD.H-2^h4^ DKO mice (Figs. 5B, 5C). Although lacrimal gland infiltration of macrophages has been reported to be regulated by CD4^+^ T cells in an SS mouse model,^87^ our data do not indicate a correlation between the prevalence of CD4^+^ T cells and macrophages (Fig. 5B). Nonetheless, the data indicate that B cells are the major infiltrates in lacrimal glands of both NOD.H-2^h4^ strains, although T helper cells likely contribute to the overall immune response. These findings are consistent with published evidence supporting the damaging effects of B cells in SS pathogenesis in humans and mouse models.^88^^–^^91^ Differences in immune infiltrates between male and female NOD.H-2^h4^ and NOD.H-2^h4^ DKO mice (Fig. 5) underscore the utility of these models for studying sexual dimorphism in human SS. The panel of antibodies used for the flow cytometry analyses did not allow for the interrogation of additional cell populations such as more specific subsets of B and T cells or CD11b^+^ antigen-presenting cells that have been reported in other murine DED models.^92^^,^^93^ Future studies will utilize a broader panel of antibodies to better understand the immunological mechanisms that mediate different sex- and strain-specific DED phenotypes in these mice.

Proinflammatory marker expression correlates with the degree of inflammation in lacrimal glands of SS mouse models, including NOD strains.^53^^,^^54^^,^^94^^,^^95^ IL-2, IL-6, TNF-α, and ICAM-1 levels were significantly higher in lacrimal glands of female and male NOD.H-2^h4^ DKO than NOD.H-2^h4^ mice (Fig. 6). These increases correlate with the early onset of dacryoadenitis in the lacrimal glands of these mice (Fig. 3). IL-2, BAFF, TNF-α and ICAM-1 were significantly higher in male than female NOD.H-2^h4^ mice, and IL-1β, IL-2, and IL-6 were significantly increased in NOD.H-2^h4^ DKO females relative to males, whereas ICAM-1 levels were elevated in males compared to females (Fig. 6). IL-1β was also significantly elevated in female NOD.H-2^h4^ DKO vs. female NOD.H-2^h4^ mice, consistent with a role in CD4^+^ T cell differentiation.^96^ Upregulation of proinflammatory genes can serve as disease biomarkers^97^^–^^99^ and future studies with NOD.H-2^h4^ DKO mice should evaluate the expression of other markers of autoimmune dacryoadenitis, including elevated levels of proteases,^100^ MHC II, and extracellular matrix proteins.^101^

MMP-9 is a matrix metalloprotease involved in the remodeling of the extracellular matrix that has been studied broadly in the context of inflammatory processes and fibrosis and more specifically in multiple ocular pathologies, including DED.^46^ Additionally, MMP-9 has been used as a diagnostic tool to measure ocular surface inflammation in DED, but its utility in severe SS, where patients have limited tear production and little sample for measurements of MMP-9 levels, has been questioned.^46^^,^^47^ Nevertheless, elevated MMP-9 expression and function have been reported in the lacrimal glands, tears, corneal epithelium, and conjunctiva of murine models of SS.^102^^,^^103^ In the current study, increased MMP-9 expression, particularly in the conjunctiva of NOD.H-2^h4^ DKO mice that exhibit severe DED (Fig. 2D), correlates with multiple hallmarks of DED in these mice, including reduced tear production (Fig. 4), increased dacryoadenitis (Fig. 3), corneal surface damage (Fig. 1), and increased proinflammatory cytokine expression (Fig. 6). Future studies will interrogate the mechanisms by which MMP-9 contributes to DED pathogenesis in NOD.H-2^h4^ DKO mice.

P2 receptors are important mediators of inflammatory responses in numerous tissues,^64^^,^^104^^–^^107^ including lacrimal glands.^108^ We have found that both the P2X7R and P2Y_2_R are upregulated in inflammatory diseases,^61^^,^^62^^,^^109^^,^^110^ whereupon their activation by adenosine 5ʹ-triphosphate (and UTP in the case of P2Y_2_R) contributes to immune cell responses.^37^^,^^63^^,^^105^^,^^107^^,^^109^^,^^111^^–^^113^ *P2X7R* and *P2Y_2_R* are significantly upregulated in lacrimal glands of female and male NOD.H-2^h4^ DKO mice compared to sex-matched NOD.H-2^h4^ mice (Fig. 7), which correlates with increased glandular lymphocytic foci numbers (Fig. 3). Both these receptors are expressed in multiple epithelia, including salivary gland epithelium,^62^^,^^63^^,^^105^^,^^114^^–^^116^ where they contribute to increases in inflammation,^37^^,^^63^^,^^105^^,^^111^^,^^113^ and deletion^63^ or inhibition^37^ of the P2Y_2_R or P2X7R, respectively, diminishes inflammation and enhances saliva production in the NOD.H-2^h4^ DKO mouse model of SS.^37^ Because underlying pathologies in both lacrimal and salivary glands likely share common mechanisms, it will be important to examine whether DED identified herein can be prevented by antagonism of P2Y_2_R and/or P2X7R.

In summary, NOD.H-2^h4^ and NOD.H-2^h4^ DKO mice spontaneously develop autoimmune DED with unique strain- and sex-dependent characteristics. Like other NOD strains, NOD.H-2^h4^ males acquire age-related dacryoadenitis earlier than females, although both sexes display symptoms of early-onset ocular disease. In contrast, female NOD.H-2^h4^ DKO mice display equivalent lacrimal gland inflammation as males by 10 weeks of age, with both sexes exhibiting earlier dacryoadenitis compared to NOD.H-2^h4^ mice. Thus NOD.H-2^h4^ DKO mice are unique in that they acquire severe SS-like DED in both sexes at a relatively young age, making comparisons between both NOD strains very informative for understanding the molecular basis and sex-dependent mechanisms of early-onset dacryoadenitis and loss of tear secretion. Furthermore, these mouse models are useful for comparing the pathogenesis of SS-related dry mouth and DED in a single mouse. Finally, the early onset and severity of DED symptoms in NOD.H-2^h4^ DKO mice make this an ideal model for investigating the efficacy of potential therapeutic interventions for SS before advancing them to humans.

## Supplementary Material

Supplement 1
