# Supplementary material for: Early Dry Eye Disease Onset in a NOD.H-2h4 Mouse Model of Sjögren's Syndrome
Source: Invest Ophthalmol Vis Sci. 2022 Jun 21;63(6):18. doi: 10.1167/iovs.63.6.18 (PMC9233292; doi:10.1167/iovs.63.6.18)
Supplement: Supplement 1 [file iovs-63-6-18_s001.pdf]

A

Spleen_Female

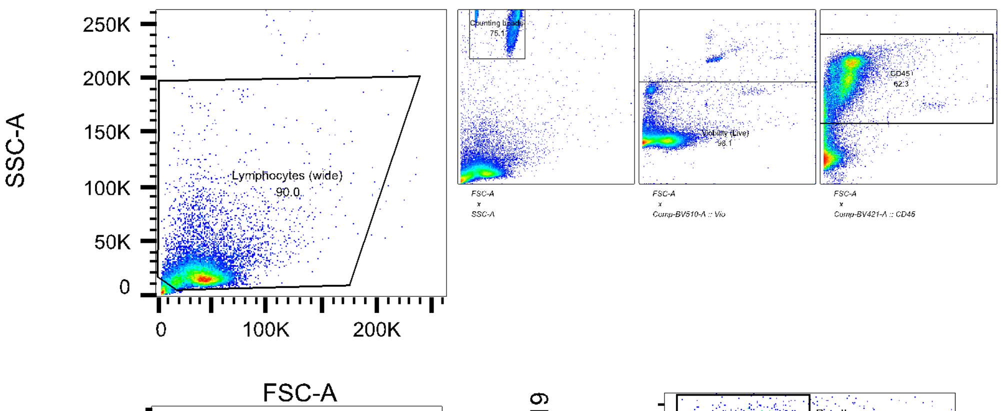

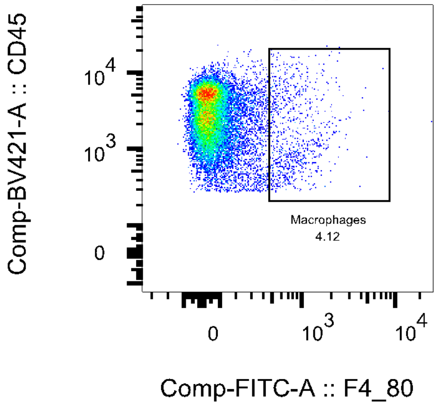

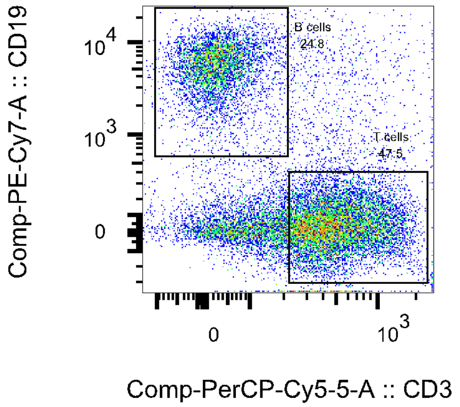

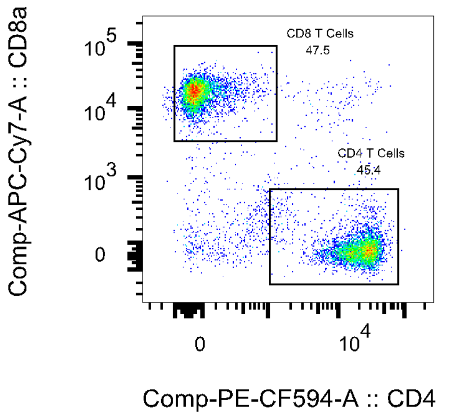

Used: 50,4000

Beads A: Count : 6742

Beads B: Count : 6651

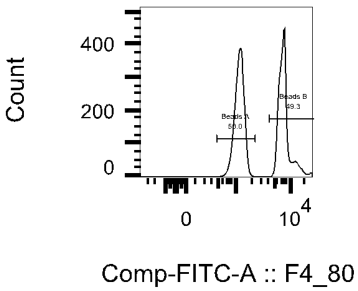

B

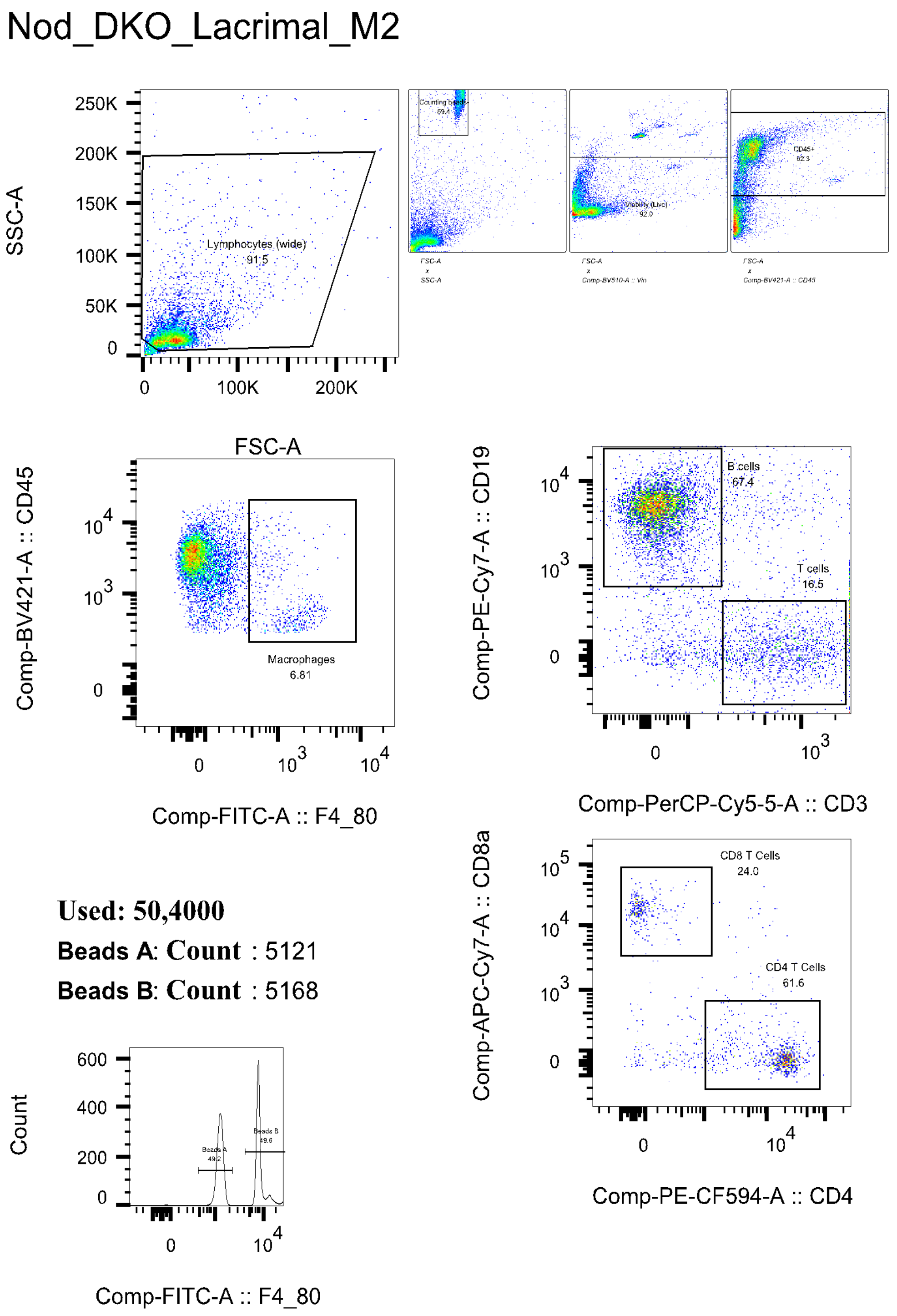

Supplementary Figure 1. Representative gating strategies for flow cytometry analysis of spleen (**A**) and lacrimal gland (**B**) samples.
